# The role of abnormal amino acid metabolism in the occurrence and development of tumors

**DOI:** 10.3389/fonc.2026.1759991

**Published:** 2026-03-05

**Authors:** Yuting Zhang, Honglin Chen

**Affiliations:** 1 Department of Pharmacy, No. 87 Zhongda Hospital Eastsouth University, Nanjing, Jiangsu, China; 2 Department of Pharmacy, Jingjiang Traditional Chinese Medicine Hospital, Jingjiang, Jiangsu, China

**Keywords:** amino acid, clinic, immunity, metabolism, tumor

## Abstract

The proliferation, migration, and invasion of tumor cells require a large amount of nutrients. Among them, the uptake of amino acids is crucial for most cellular functions, such as protein synthesis and cell growth. Tumor cells obtain a large number of essential amino acids from the environment through unique metabolic pathways and accelerate the synthesis of non-essential amino acids to meet their own needs. This review summarizes the uptake and utilization of amino acids by tumors and their inhibitory effects on immune cells, providing a basis for targeted metabolism in cancer treatment.

## 1 Introduction

Metabolic reprogramming is a well-established hallmark of cancer, enabling tumor cells to adapt to the dynamic and often hostile conditions of the tumor microenvironment (TME). This rewiring of cellular metabolism supports rapid proliferation, survival under stress, and evasion of immune surveillance by altering nutrient availability and signaling networks (1). Among the various metabolic pathways dysregulated in cancer—such as glycolysis, lipid metabolism, and mitochondrial respiration—amino acid metabolism has emerged as a particularly critical node that intersects tumor biology and immunomodulation (2). Unlike other metabolic alterations that primarily fuel bioenergetic demands, reprogrammed amino acid metabolism exerts dual effects: it directly promotes tumor growth and metastasis while simultaneously shaping the functional states of immune cells within the TME.

The amino acids correspond to the six critical aspects of tumor immunometabolism:

Energy and Biosynthetic Precursors (Glutamine, Serine): Glutamine serves as a central donor of nitrogen and carbon, playing a pivotal role in TCA cycle anaplerosis and nucleotide synthesis. Serine, on the other hand, acts as a hub for one-carbon metabolism, directly linking the folate cycle to the synthesis of purines and pyrimidines. Together, they address how cells “build the bricks and mortar” and “supply the fuel” for rapid proliferation.

Immunoregulation and Microenvironmental Signaling (Tryptophan, Arginine): The depletion of tryptophan via the kynurenine pathway and the accumulation of its metabolites represent classic paradigms mediating T cell dysfunction and the formation of an immunosuppressive microenvironment. Arginine metabolism directly regulates macrophage polarization (M1 *vs*. M2) as well as T cell activation and proliferation. These amino acids illustrate how metabolites can directly function as immune checkpoints and cell fate regulators.

Redox Homeostasis and Adaptive Survival (Methionine, Proline): The methionine cycle profoundly influences the cellular epigenetic state and antioxidant capacity by modulating methylation reactions and glutathione synthesis. Proline metabolism, associated with oxidative stress response and collagen synthesis, is linked to the construction of the tumor metastatic microenvironment. They explain how cells achieve environmental adaptation and survival defense through metabolism.

The depletion or accumulation of specific amino acids (e.g., tryptophan, glutamine, arginine), coupled with the upregulation of key metabolic enzymes (such as IDO1, ARG1, and ASCT2) and amino acid sensors (e.g., GCN2, mTOR), actively suppresses anti-tumor immunity by impairing T cell activation, promoting regulatory T cells, and polarizing myeloid cells toward an immunosuppressive phenotype (3). For example, indoleamine 2,3-dioxygenase 1 (IDO1)-mediated tryptophan catabolism not only limits T cell proliferation through amino acid deprivation but also generates immunosuppressive kynurenine metabolites that activate the aryl hydrocarbon receptor (AhR) in immune cells (4). Similarly, arginine consumption by myeloid-derived suppressor cells (MDSCs) via arginase 1 (ARG1) leads to T cell dysfunction and cell cycle arrest (5). Given its central role in both tumor progression and immune escape, targeting amino acid metabolism offers a promising therapeutic strategy to overcome resistance to current immunotherapies, such as PD-1/PD-L1 blockade, which currently benefit only a subset of patients (response rates ~20–40%) (6). Thus, understanding the interplay between amino acid metabolic reprogramming and immune dysfunction in the TME is crucial for developing more effective combination therapies.

## 2 Abnormal amino acid metabolism promotes the growth of tumor cells

### 2.1 Glutamine

Glutamine (Gln) is the most abundant free amino acid in human circulation and is crucial for rapidly proliferating tumor cells. Unlike normal cells, many cancer cells exhibit a phenomenon known as “glutamine addiction,” consuming it at rates far exceeding biosynthetic demands. The role of glutamine extends beyond protein synthesis; it drives central carbon metabolism, nitrogen metabolism, maintains redox homeostasis, regulates signaling pathways, and promotes immune evasion. Reprogramming of glutamine metabolism is thus a key hallmark of tumor metabolic remodeling (7, 8).

Tumor cells primarily utilize glutamine through two pathways: oxidative metabolism via the tricarboxylic acid (TCA) cycle to sustain energy supply under aerobic glycolysis (Warburg effect) conditions, and reductive carboxylation under hypoxia or mitochondrial dysfunction, where glutamine-derived α-ketoglutarate (α-KG) serves as a carbon source for citrate and lipid biosynthesis. Glutamine-derived α-KG also enhances antioxidant defense: it promotes fumarate production, which subsequently upregulates glutathione peroxidase 1 (GPx1), scavenging reactive oxygen species (ROS) and protecting tumor cells from oxidative stress (9). Moreover, glutamine contains two nitrogen atoms and provides nitrogen for the synthesis of purines and pyrimidines (such as DNA/RNA), which is a key raw material for nucleotide formation. It also participates in the synthesis of non-essential amino acids, promoting protein synthesis, meeting the growth needs of tumors and providing nitrogen for the synthesis of polyamines that promote proliferation, as well as antioxidant glutathione, enhancing the survival ability of tumor cells (10).

First, regarding the role of metabolic enzymes, glutamine is initially hydrolyzed into glutamate in the mitochondria by glutaminase (GLS), with the GLS1 isoform predominantly driving this process in most tumors. Although both GLS1 and GLS2 catalyze the conversion of glutamine to glutamate, they exhibit significant differences in expression patterns, regulatory mechanisms, and biological functions, displaying context-dependent functional opposition.

GLS1 is widely overexpressed in rapidly growing tumors and is directly transcriptionally activated by oncogenes such as c-Myc—c-Myc binds to the promoter region of the GLS gene to induce its expression (11). This upregulation meets the energy and material demands of cell proliferation, making GLS1 a typical pro-oncogenic factor. In various tumor models, inhibiting GLS1 effectively suppresses tumor growth, highlighting its broad functional importance.

In contrast, GLS2 plays a dual role with tumor-suppressive potential. It is a direct transcriptional target of the tumor suppressor protein p53 and is primarily expressed in differentiated tissues such as the liver (12). Under physiological conditions, GLS2 reduces ROS levels by maintaining mitochondrial integrity and promoting glutathione (GSH) synthesis, fulfilling the conserved stress-responsive function of p53.

However, in hepatocellular carcinoma (HCC), GLS2 is often silenced due to promoter hypermethylation or inactivation of wild-type p53. Surprisingly, restoring GLS2 expression inhibits tumor growth both *in vitro* and in animal models. This tumor-suppressive effect has recently been attributed to an iron-dependent form of programmed cell death—ferroptosis. Mechanistically, GLS2 overexpression elevates intracellular glutamate levels, which competitively inhibits the cystine/glutamate antiporter system xc^−^ (composed of SLC7A11/xCT), reduces cystine uptake, depletes GSH, and impairs GPX4s ability to clear lipid peroxides. For HCC cells already under oxidative stress, this disturbance disrupts redox homeostasis, triggers lethal lipid peroxidation, and ultimately induces ferroptosis (13). Thus, GLS2 acts as a “double-edged sword”: protective in normal or mildly stressed cells but potentially tumor-suppressive in transformed hepatocytes. It is important to emphasize that the function of GLS2 is highly tissue-context-dependent. In glioma and certain breast cancer models, GLS2 has been reported to promote stemness maintenance or mediate therapy resistance, for example, by activating the mTOR signaling pathway. This indicates that metabolic enzymes cannot be simply categorized as “pro-oncogenic” or “tumor-suppressive”; their roles must be evaluated in the context of specific pathological backgrounds, genetic states, and overall metabolic networks.

Beyond metabolic enzymes, changes in transporter proteins also play a significant role in shaping glutamine metabolism in the tumor microenvironment. Since tumor cells cannot synthesize sufficient glutamine *de novo*, they must rely heavily on exogenous uptake. To this end, cancer cells upregulate specific amino acid transporters to enhance nutrient absorption and activate downstream signaling pathways. The neutral amino acid transporters ASCT2 (SLC1A5), LAT1 (SLC7A5), and LAT2 (SLC7A8) are consistently overexpressed in various malignancies, including breast, pancreatic, and lung cancers, serving as the primary entry points for glutamine, leucine, methionine, and other key nutrients (14).

Among these, ASCT2 and LAT1 function in a coupled manner to activate mTORC1. ASCT2 is the main channel for glutamine uptake in tumor cells, transporting extracellular glutamine into cells via Na^+^-dependent cotransport. More importantly, as an antiporter, it releases intracellular amino acids (such as leucine, serine, and alanine) into the extracellular space while importing glutamine (15). These extruded amino acids serve as substrates for LAT1, driving the uptake of essential branched-chain and aromatic amino acids (e.g., leucine). LAT1 functions as an antiporter, importing essential amino acids like leucine in exchange for exporting intracellular glutamine.

Leucine is a potent activator of mTORC1, a master regulator of protein synthesis, cell growth, and metabolism. Once activated, mTOR signaling in turn promotes the expression of ASCT2 and LAT1, forming a positive feedback loop (ASCT2–LAT1–mTOR) that continuously drives tumor proliferation and metabolic adaptation (16).

This transporter coupling not only supports biosynthesis but is also closely linked to epigenetic regulation. For instance, in lung cancer, the oncogene IGF2BP2 stabilizes LAT1 mRNA, increasing methionine uptake. Methionine is a precursor of S-adenosylmethionine (SAM), which serves as a methyl donor for the histone methyltransferase SETD1A, catalyzing trimethylation of histone H3 at lysine 4 (H3K4me3). This activating chromatin mark further enhances IGF2BP2 transcription, establishing a self-reinforcing Met/SAM/H3K4me3–IGF2BP2–LAT1 positive feedback circuit that exacerbates tumor progression (17).

Additionally, studies have shown that knocking down LAT1 significantly inhibits the viability, migration, and invasion of oxaliplatin-resistant gastric cancer cells and slows tumor growth *in vivo*. This is accompanied by upregulation of the pro-apoptotic protein BAX, downregulation of the anti-apoptotic protein BCL2, and reduced expression of metastasis-related proteins such as MMP2 and MMP9. Metabolically, LAT1 deficiency leads to decreased expression of key glycolytic enzymes HK2, LDHA, Glut1, and PDK1, suggesting its role in regulating energy metabolism via the mTOR pathway (18).

Meanwhile, LAT2 (SLC7A8), long considered less significant than ASCT2 or LAT1, has recently emerged as a key player in pancreatic cancer progression and immune regulation (18), becoming a novel regulatory node in tumor-immune interactions.

Clinical data indicate that high LAT2 expression is significantly correlated with decreased overall survival in pancreatic ductal adenocarcinoma (PDAC) patients. LAT2 can be upregulated by IL-18 secreted by tumor-associated macrophages, enhancing tumor cell uptake of glutamine and leucine. This process activates the mTOR signaling pathway and subsequently upregulates CD47—the “don’t eat me” signal—thereby inhibiting macrophage phagocytosis and facilitating immune escape (19). Further research reveals that LAT2 physically interacts with phosphorylated mTOR (p-mTOR Ser2448) and maintains intracellular glutamine accumulation through a positive feedback loop involving “LAT2/p-mTOR/glutamine synthetase (GS).” Concurrently, LAT2 downregulates glutaminase (GLS) expression, inhibiting glutamine catabolism and further elevating intracellular glutamine levels. Activated mTOR signaling upregulates lactate dehydrogenase B (LDHB), promoting glycolysis and reducing sensitivity to chemotherapy drugs such as gemcitabine (GEM) (20). Notably, genetic or pharmacological inhibition of LAT2 can reverse the IL-18-induced suppression of phagocytosis, restore macrophage-mediated tumor clearance, and enhance anti-tumor immunity (21). These findings indicate that LAT2 is not merely a metabolic regulator but also a critical nexus in the interaction between tumor cells and immune components within the tumor microenvironment.

### 2.2 Serine and glycine

More than half a century ago, scientists noticed abnormal changes in serine metabolism within tumor cells. The most significant feature was a significant increase in the metabolic flow of the serine *de novo* synthesis pathway (SSP). This pathway branches off from the glycolytic intermediate 3-phosphoglycerate and requires the catalysis of three enzymes in sequence: 3-phosphoglycerate dehydrogenase (PHGDH), phosphoserine aminotransferase (PSAT1), and phosphoserine phosphatase (PSPH) (22). Through the action of serine hydroxymethyltransferase (cytosolic SHMT1 or mitochondrial SHMT2), serine can be further converted into glycine, providing a carbon unit for the folate cycle and ultimately used for the biosynthesis of nucleotides. As two amino acids closely related to biosynthesis, serine and glycine are important precursors for protein, nucleic acid, and lipid synthesis in tumor proliferation.

The research has found that mitochondrial SHMT2 is one of the most frequently overexpressed “metabolic genes” in human tumors. As a key enzyme for the conversion of serine to glycine, its knockout can significantly inhibit the proliferation of tumor cells. SHMT2 promotes the growth of tumors in hypoxic environments by maintaining low serine concentrations within the cells (23). Studies have shown that serine acts as a co-activator for the M2-type pyruvate kinase (PKM2). When its concentration decreases, the activity of PKM2 is inhibited, leading to the accumulation of glycolytic intermediates. This regulation limits the entry of pyruvate into the TCA cycle, thereby reducing the production of reactive oxygen species, providing a survival advantage for glioma cells in hypoxic conditions. Under normal conditions, inhibiting SHMT2 does not seem to affect the survival activity of cells. However, in hypoxic or nutrient-limited conditions, SHMT2 is a key determinant of tumor cell survival. After inhibiting SHMT2 activity, in addition to the expected accumulation of serine and depletion of glycine, it can also increase the levels of TCA cycle intermediates, reduce the level of the pentose phosphate pathway, increase oxygen consumption, and promote the entry of NADH into the oxidative phosphorylation pathway. Moreover, this pro-survival effect of SHMT2 depends on the clearance of amino-pyrone and methylglyoxal, toxic metabolites of glycine, by the glycine decarboxylase (GLDC) (24). GLDC is overexpressed in some human cancers and is one of the most significantly upregulated genes in tumor cells isolated from non-small cell lung cancer. Because serine can mediate drug resistance through various mechanisms such as maintaining intracellular antioxidant capacity or continuously providing raw materials for nucleotide synthesis, therapeutic strategies targeting serine metabolism have shown potential in inhibiting tumor growth and enhancing the efficacy of anti-cancer drugs (25).

PHGDH is a key rate-limiting enzyme in the serine synthesis pathway, catalyzing the conversion of 3-phosphoglyceric acid to 3-phosphohydroxypropionic acid. In various cancers such as lung cancer, colorectal cancer, pancreatic cancer, breast cancer, and liver cancer, the expression of PHGDH is upregulated, and it is associated with tumor stage, metastasis, and poor prognosis (26). By promoting the “one-carbon unit” metabolism of serine, PHGDH provides sufficient raw materials for the DNA replication and RNA transcription of tumor cells, serving as the material basis for their rapid proliferation (27). Tumor cells produce a large amount of reactive oxygen species (ROS) during rapid growth and under drug treatment, and PHGDH helps them resist this oxidative stress. Through the one-carbon metabolism and transsulfur pathway, the metabolic products of serine contribute to the synthesis of glutathione, thereby enhancing the ability of tumor cells to eliminate ROS (28). Multiple steps in the serine catabolism pathway (such as the reaction catalyzed by MTHFD2) can generate NADPH. NADPH is a cofactor of glutathione reductase, used to reduce oxidized glutathione and regenerate its antioxidant function. High expression of PHGDH enables tumor cells to have a stronger “antioxidant shield”, not only resisting endogenous oxidative stress but also weakening the effects of chemotherapy (such as doxorubicin) and radiotherapy that kill tumors by generating ROS, thereby leading to treatment resistance (29). For example, in human colorectal cancer cell lines, the hypoxic environment increases the expression of PHGDH. The inhibitory effect of PHGDH will increase the level of reactive oxygen species (ROS), thereby enhancing radiosensitivity (30). In non-small cell lung cancer (NSCLC), the expression of PHGDH in tumor tissues is higher than that in matched adjacent lung tissues. Moreover, using one-step quantitative reverse transcription polymerase chain reaction (qRT–PCR) detection, it was found that the PHGDH mRNA level in human NSCLC tissues is higher than that in adjacent normal tissues. Immunohistochemical analysis shows that PHGDH expression is positively correlated with TNM (tumor status, lymph node metastasis, distant metastasis) staging, which indicates that this increase is consistent with the evidence that patients with high PHGDH levels have shorter survival times than those with low PHGDH levels, suggesting that PHGDH may be involved in the cancer progression of NSCLC patients (31).

### 2.3 Proline

Proline, as a structurally unique non-essential amino acid, has a special metabolic pathway for its biosynthesis. It can be generated through the ornithine pathway or converted from glutamate. Both of these synthesis pathways will produce the key intermediate product L-glutamate-γ-hydroxy acid (GSAL), which will spontaneously cyclize under dehydration conditions to form P5C (32). Studies have found that the mutual conversion between proline and glutamate is achieved through intermediates such as P5C and GSA. Particularly noteworthy is that the proline dehydrogenase induced by p53 and PPARγ can catalyze the conversion of proline to P5C, and this process plays an important role in anti-cancer functions in mitochondria (33). As another part of the metabolic cycle, GSA derived from glutamate or proline can be reconverted into ornithine to maintain metabolic balance.

During the process of tumor occurrence and development, proline metabolism exhibits multiple regulatory effects. As a secondary amino acid with a unique structure in protein synthesis, proline has a complete independent metabolic pathway. In different subcellular compartments, members of the PYCR enzyme family show significant localization differences: PYCR1 and PYCR2 are mainly distributed in mitochondria and tend to utilize NADH as a cofactor; while PYCR3 is located in the cytoplasm and is more dependent on NADPH (34). This compartmentalized distribution provides a structural basis for the precise regulation of proline metabolism. Studies in the tumor microenvironment have found that PYCR1 expression is frequently upregulated in breast cancer-associated fibroblasts (CAFs). This upregulation is closely related to the synthesis level of collagen. When PYCR1 is inhibited through genetic or drug means, ribosomes will be stalled on collagen mRNA, thereby significantly reducing collagen production. Further animal experiments have confirmed that tumor cells co-transplanted with PYCR1-deficient CAFs exhibit reduced collagen deposition, delayed growth, and restricted metastasis (35). These findings highlight the crucial role of proline metabolism in tumor matrix remodeling.

In the research on esophageal squamous cell carcinoma (ESCC), it was found that the overexpression of the ORAOV1 gene may promote proline synthesis by binding and activating PYCR. This process will increase the intracellular proline level while reducing the production of reactive oxygen species (ROS), thereby creating favorable conditions for tumor progression. Moreover, proline metabolism also affects the fate of tumor cells through its unique redox regulatory function. During synthesis, it consumes NAD(P)H to produce NAD(P)^+^; while during decomposition, it can regenerate NAD(P)H. This reversible transformation makes proline metabolism an important hub for maintaining the cellular redox balance, especially playing an irreplaceable role in tumor microenvironments with elevated ROS levels and hypoxia (36, 37). These research findings not only deepen our understanding of the complexity of proline metabolism, but also provide an important theoretical basis for the development of new anti-tumor therapies.

### 2.4 Arginine

Arginine, as a conditionally essential amino acid, plays a crucial role under specific pathological and physiological conditions. Arginine succinate synthetase (ASS) catalyzes the synthesis of arginine succinate from L-citrulline and aspartate. This reaction is the rate-limiting step in the *de novo* synthesis of arginine. Subsequently, arginine succinate lyase (ASL) breaks down arginine succinate into L-arginine and fumaric acid, which is coupled with the metabolism of arginine and glucose energy metabolism through the TCA cycle. It is noteworthy that arginine metabolic disorders caused by ASS deficiency are common features of aggressive tumors, especially malignant melanoma and hepatocellular carcinoma (38).

Molecular-level studies have revealed that ASS1 expression is absent in various tumors, including osteosarcoma, making their growth completely dependent on exogenous arginine. Clinical data show that patients with ASS1-deficient osteosarcoma are more prone to lung metastasis and have a poorer prognosis. This invasive phenotype is closely related to the increased activity of arginase (ARG1/2) in the tumor microenvironment. In breast cancer and gastric cancer, the enhanced activity of ARG1 promotes the expansion of tumor-associated myeloid cells (TAMCs). These cells respond to tumor-derived factors such as M-CSF, IL-4, IL-6, TGF-β, GM-CSF, and HIF-1α-related pathways, forming a positive feedback loop with increased ARG1 expression. The breakdown products of arginine not only provide precursors for the synthesis of polyamines in tumor cells but also promote tumor progression by reshaping the immune microenvironment (39) (**Table 1**).

**Table 1:** The effects of five amino acids on the key upregulated enzymes/transports in tumors and on the biology of tumor cells.

Amino acid	Key upregulated enzymes/transport proteins	The impact on the biology of tumor cells
Glutamine	GLS1 (glutaminase 1), ASCT2 (SLC1A5), LAT1 (SLC7A5), LAT2 (SLC7A8)	Activates TCA cycle for energy replenishment, nucleic acid synthesis, lipid synthesis, antioxidant (GSH), collagen deposition; supports the "glutamine addiction" phenotype; GLS2 has a tumor-suppressing effect in liver cancer, achieved by inducing ferroptosis (inhibiting system xc^−^ → depletion of GSH → inactivation of GPX4 → lipid peroxidation); High expression of PYCR1 promotes ECM hardening and metastasis
Serine	PHGDH, PSAT1, PSPH (serine de novo synthesis pathway enzymes), SHMT2 (mitochondrial serine hydroxymethyltransferase)	Provide one-carbon units for nucleotide (purine/pyrimidine) synthesis; Maintain redox homeostasis (through NADPH generation and GSH synthesis); Enhance resistance to radiotherapy and chemotherapy; High expression of PHGDH is positively correlated with tumor stage, metastasis, and poor prognosis; In hypoxia, SHMT2 maintains low intracellular serine concentration, supporting survival
Proline	PYCR1, PYCR2 (proline synthase, located in mitochondria), PRODH (proline dehydrogenase, catalyzing decomposition)	PYCR1 drives collagen deposition and ECM hardening, promoting invasion and metastasis; PRODH produces ROS, promoting the secretion of inflammatory factors; Regulates redox balance in both directions, supporting hypoxia adaptation; Proline metabolism participates in epigenetic regulation (such as SAM supply)
Arginine	ARG1 (Arginase 1), ASS1 deficiency (dependent on exogenous factors), SLC7A5/SLC3A2 (LAT1/CD98hc)	Increased ARG1 activity provides precursors of polyamines and promotes proliferation; The absence of ASS1 remodels the immune microenvironment; Disorder of arginine metabolism is closely related to invasion, metastasis and poor prognosis
Tryptophan	IDO1 (Indoleamine 2,3-dioxygenase 1), TDO2 (Tryptophan 2,3-dioxygenase), SLC7A5/SLC3A2 (LAT1/CD98hc)	IDO1/TDO2 catalyzes the degradation of tryptophan into kynurenine, consuming the local tryptophan environment; Kynurenine activates AhR, promoting tumor progression and immune evasion

## 3 The impact of abnormal amino acid metabolism on tumor immunity

In summary, tumor cells reprogram amino acid metabolism to meet the demands of rapid proliferation, antioxidant defense, and epigenetic regulation, demonstrating a strong dependence on and aberrant utilization of specific amino acids (such as glutamine, tryptophan, arginine, etc.). However, the impact of these metabolic alterations extends far beyond the cancer cells themselves—the resulting nutrient deprivation, accumulation of metabolites, and changes in the pH and redox state of the microenvironment profoundly reshape the function and fate of surrounding immune cells. Indeed, competition between tumor cells and immune cells for limited resources, coupled with metabolite-mediated signaling interference dominated by cancer cells, has become a key driver in the formation of an immunosuppressive tumor microenvironment. Within this context, the amino acid metabolism of tumor-infiltrating immune cells inevitably undergoes adaptive or suppressive remodeling, further influencing their activation, differentiation, and effector functions. Therefore, understanding how immune cells respond and adapt to this “hijacked” metabolic ecology in the tumor microenvironment represents a critical entry point for uncovering the mechanisms of cancer immune evasion and developing novel immunometabolic intervention strategies.

### 3.1 Glutamine

In the immune system, glutamine is not only an essential amino acid nutrient but also a key metabolic substrate that regulates the function of immune cells (40). T cells in peripheral blood circulation are typically in a resting state, with their energy metabolism dominated by oxidative phosphorylation and overall low metabolic activity. However, when these circulating T cells are stimulated by antigens and activated through the TCR signaling pathway, they rapidly initiate a vigorous metabolic reprogramming process (41). During this process, the uptake and catabolism of glutamine are significantly enhanced, serving as a crucial foundation for the rapid proliferation, biosynthesis, and effector molecule expression of T cells (3). Studies have shown that the ability of activated T cells to uptake glutamine through the ASCT2 transporter is five to ten times higher than that in the resting state, demonstrating a strong metabolic dependency. Once inside the cell, glutamine is converted into glutamate by glutaminase and further participates in the replenishment of tricarboxylic acid cycle intermediates, nucleotide synthesis, and the generation of reduced glutathione, thereby maintaining a high level of aerobic glycolysis and antioxidant capacity. This metabolic shift not only provides T cells with sufficient energy and building materials but also influences their differentiation fate - promoting the development of pro-inflammatory Th1 and Th17 cells while inhibiting the generation of regulatory T cells with immunoregulatory functions, thus playing a decisive role in the overall intensity of the immune response (42, 43).

In contrast, in the tumor microenvironment, the metabolic environment in which tumor-infiltrating immune cells reside is more complex and challenging. Although T cells here have the ability to recognize tumor antigens, their functions are often severely weakened due to nutrient deprivation, accumulation of metabolic waste, and the presence of inhibitory signals (44). Cancer cells themselves have a strong dependence on glutamine and widely express ASCT2 and glutaminase, consuming a large amount of glutamine in the local microenvironment, resulting in a shortage of available resources for immune cells (45). At the same time, tumor-associated cells are also actively involved in this metabolic competition. For instance, tumor-associated neutrophils can secrete lipocalin-2, which can enhance the localization efficiency of ASCT2 on the T cell membrane, theoretically helping to increase the uptake of glutamine (46). However, in most solid tumors, this potential positive regulation is often masked by lactic acid accumulation, hypoxia, and co-inhibitory signals from immune checkpoint molecules (47). Single-cell sequencing studies have revealed that some tumor-infiltrating T cell populations still retain a certain degree of metabolic plasticity, especially those T cell subpopulations with memory precursor characteristics, which have a relatively high utilization efficiency of glutamine, suggesting that intervention in their metabolic pathways may restore their anti-tumor activity (48).

In addition to T cells, tumor-infiltrating macrophages, as key regulators in the microenvironment, have their polarization states profoundly influenced by glutamine metabolism (49). M2-type macrophages exhibit a significantly higher glutamine catabolic flux than M1-type macrophages, a difference mainly attributed to the specific high expression of the mitochondrial glutamate carrier SLC25A22, which enables glutamate to efficiently enter mitochondria to participate in energy and intermediate metabolite production (50). M2-type macrophages rely on this metabolic pathway to support the activity of arginase-1 and the synthesis of molecules such as polyamines that promote tissue repair and angiogenesis, thereby exerting immunosuppressive and pro-tumor growth effects (51, 52). Recent studies have found that selectively inhibiting the GLS2 subtype of glutaminase can promote the transformation of tumor-associated macrophages to an M1-like phenotype without significantly affecting the metabolism of normal tissues, characterized by increased secretion of pro-inflammatory factors and weakened immunosuppressive function (53). This metabolic intervention strategy has shown promising anti-tumor effects in mouse models of pancreatic cancer and colorectal cancer and can significantly enhance the efficacy of PD-1 blockade therapy, suggesting its great potential in combination immunotherapy (54).

Further in-depth research has revealed a complex bidirectional metabolic dialogue between tumor cells and tumor-infiltrating immune cells (55). Tumor cells can release extracellular vesicles carrying glutamine synthetase to re-synthesize glutamine from accumulated glutamate in the environment, dynamically regulating the local amino acid composition and thereby influencing the functional state of immune cells (56). Moreover, metabolic products themselves have been found to have signaling functions. For instance, α-ketoglutarate derived from glutamine serves as a cofactor for various epigenetic modification enzymes, promoting DNA demethylation and facilitating the differentiation of anti-tumor T cells; while other metabolites such as succinate and fumarate stabilize HIF-1α to promote Th17 polarization or enhance immunosuppression (57). Novel metabolic sensor molecules such as SIRT4 and NAD^+^-dependent deacetylases have also been confirmed to participate in regulating the mitochondrial function and fate determination of immune cells, providing new molecular targets for precise intervention (58, 59).

Against this backdrop, drugs targeting glutamine metabolism are emerging as a crucial approach to improving the tumor immune microenvironment (60). The GLS1 inhibitor Telaglenastat (CB-839) has demonstrated the ability to alleviate the excessive consumption of glutamine by cancer cells in various tumor models, reduce metabolic suppression on T cells, and restore their functional activity (61). Preclinical studies have confirmed that this drug not only reduces the accumulation of myeloid-derived suppressor cells but also enhances the infiltration and effector function of CD8^+^ T cells (62). Currently, multiple clinical trials are evaluating the safety and efficacy of Telaglenastat in combination with PD-1 antibodies in patients with renal cell carcinoma and non-small cell lung cancer. Preliminary results indicate that the tumor microenvironment in some patients has shifted from “cold” to “hot”, suggesting that the immune response has been effectively activated (63, 64).

In summary, circulating immune cells and tumor-infiltrating immune cells exhibit strikingly different characteristics in terms of glutamine metabolism: the former reflects the systemic immune activation requirements, while the latter demonstrates the difficult balance between survival and functional realization in a highly competitive and suppressive microenvironment (44). Understanding the metabolic differences between these two types of cells and their interaction mechanisms not only helps to reveal the essence of tumor immune escape but also provides a solid theoretical basis and feasible path for the development of novel combined immunotherapy strategies based on metabolic regulation (65, 66). With the advancement of spatial metabolomics and single-cell multi-omics technologies in the future, we will be able to more precisely depict the metabolic profiles of various immune cells in the tumor microenvironment, propelling personalized and precise cancer immunotherapy into a new stage (67).

### 3.2 Tryptophan

In the tumor microenvironment, the disruption of tryptophan metabolism is one of the core strategies for tumors to achieve immune evasion. This process begins with the high expression of indoleamine 2,3-dioxygenase (IDO) in tumor cells, which consumes a large amount of tryptophan in the microenvironment. To survive in the environment lacking tryptophan created by themselves, IDO-positive tumor cells have evolved a unique adaptive mechanism: they upregulate a highly specific tryptophan transporter: SLC7A5/SLC3A2 heterodimer (also known as LAT1/CD98hc). This transporter does not rely on sodium ions and is not inhibited by glutamine competitively, thus ensuring that in the case of widespread depletion of tryptophan, tumor cells can still efficiently and exclusively take up tryptophan, while further exacerbating the depletion of tryptophan in the microenvironment, causing T cells dependent on tryptophan to fall into a deep state of “nutritional starvation” (68, 69).

When intracellular tryptophan levels decline, tryptophanyl-tRNA synthetase (TrpRS), the aminoacyl-tRNA synthetase responsible for charging tRNA^Trp with its cognate amino acid, lacks its substrate and thus fails to catalyze the aminoacylation of tRNA^Trp. This results in the accumulation of uncharged tRNA^Trp in the cytoplasm. These uncharged tRNAs are not merely metabolic byproducts but function as critical signaling molecules that actively regulate cellular stress pathways. General control nonderepressible 2 (GCN2), a serine/threonine kinase, contains a highly conserved histidyl-tRNA synthetase-like domain (HisRS-like domain) that shares structural homology with canonical aminoacyl-tRNA synthetases. This domain enables GCN2 to specifically recognize and bind various uncharged tRNAs, including uncharged tRNA^Trp (70). Binding of uncharged tRNA induces a conformational change in GCN2, relieving its autoinhibitory state and exposing its kinase domain, thereby triggering autophosphorylation and full activation (71).

Activated GCN2 phosphorylates the α subunit of eukaryotic initiation factor 2 (eIF2α) at serine 51. Phosphorylated eIF2α (p-eIF2α) can still assemble into the 43S pre-initiation complex but exhibits impaired nucleotide exchange due to reduced interaction with eIF2B, the guanine nucleotide exchange factor (72). This leads to a significant decrease in global translation initiation and widespread suppression of protein synthesis. Importantly, this translational inhibition is not uniform across all transcripts. mRNAs containing upstream open reading frames (uORFs) in their 5’ untranslated regions are preferentially translated under these conditions. A prototypical example is activating transcription factor 4 (ATF4), whose translation is selectively enhanced when eIF2α is phosphorylated (73). As a master regulator of the integrated stress response (ISR), ATF4 subsequently upregulates a panel of stress-responsive genes, including CHOP (DDIT3), GADD34 (PPP1R15A), and amino acid transporters, thereby orchestrating adaptive responses to nutrient deprivation (74).

In T lymphocytes, activation of the GCN2 pathway not only disrupts metabolic homeostasis but also profoundly influences cell proliferation and differentiation fate. Persistent eIF2α phosphorylation has been shown to induce G1-phase cell cycle arrest, thereby suppressing clonal expansion of T cells. Moreover, ATF4-dependent signaling interferes with the expression of RORγt, the lineage-defining transcription factor for Th17 cells, leading to impaired development of pro-inflammatory T helper 17 (Th17) cells. Concurrently, this pathway promotes the differentiation of immunosuppressive Foxp3-expressing regulatory T cells (Tregs) (75). This shift in CD4^+^ T cell differentiation balance fosters a tolerogenic immune environment—beneficial for maintaining self-tolerance under physiological conditions but exploited in pathological contexts such as the tumor microenvironment to facilitate immune evasion.

This complex molecular mechanism provides new targets for cancer immunotherapy. Studies have shown that targeting the intervention of the IDO-AhR signaling axis can effectively reverse the functional exhaustion of T cells, especially when combined with traditional immune checkpoint inhibitors, which can significantly enhance the anti-tumor immune response, laying the theoretical foundation for the development of new combined treatment regimens (76). Although the first IDO1 inhibitor, Epacadostat, failed in the III-phase clinical trial, this result prompted scientists to conduct in-depth reflection. The current research direction has shifted to developing dual inhibitors that can simultaneously inhibit IDO1 and TDO2, or directly target the downstream AhR receptor (such as the AhR antagonist IK-175), in order to more comprehensively and thoroughly block this immunosuppressive pathway. In addition, detecting the level of tryptophan metabolism in the blood, such as calculating the ratio of kynurenine to tryptophan, is expected to become a potential biomarker for predicting the efficacy of immunotherapy and screening out individuals with superior benefits in the future (77).

### 3.3 Arginine

Arginine plays a dual role in the regulation of T cell immune function. As a crucial immune metabolic regulatory molecule, arginine can activate specific gene expression programs, significantly increase the energy metabolism level of T cells, promote the formation of central memory T cells, and thereby enhance anti-tumor immune activity. However, abnormal changes in arginine metabolism in the tumor microenvironment often weaken these positive effects (78).

Argininease (Arg1) is stored in the cytoplasmic granules of neutrophils. After being spontaneously activated *in vitro*, neutrophils release the active arginine into the extracellular environment (79). Under pathological conditions, the abnormal increase in the activity of argininease (ARG1) accelerates the catabolism of arginine. The nitric oxide (NO) produced during this process not only inhibits the proliferation ability of T cells but also induces T cell apoptosis by suppressing the expression of major histocompatibility complex II (MHC-II) molecules. What is more complex is that the arginine depletion mediated by ARG1 can also lead to uncoupling of nitric oxide synthase (NOS), thereby generating superoxide anion (O^2-^). Under the special pathological conditions of the tumor microenvironment, NO combines with O^2-^ to form various reactive nitrogen species (RNS), and these highly reactive molecules further damage the functional integrity of T cells (80).

In-depth studies have shown that this imbalance in arginine metabolism constitutes an important mechanism for tumor immune evasion. By creating a microenvironment lacking arginine, tumor cells not only directly suppress the anti-tumor function of T cells but also indirectly affect the activity of other immune cells. This multi-level immune suppression network provides important targets for the development of new immunotherapy strategies. The latest preclinical experiments have demonstrated that arginine metabolism intervention combined with immune checkpoint blockade can significantly enhance the anti-tumor effect, providing a new idea for overcoming the current problem of immune therapy resistance (81).

The early clinical trial of the arginase inhibitor CB-1158 demonstrated that, whether used as a single drug or in combination with immune checkpoint inhibitors, it could significantly restore the activity of CD8^+^ T cells, and objective responses were observed in some patients with solid tumors. Additionally, arginine supplementation strategies (such as recombinant human arginase 1 inhibitor) are also being explored, aiming to reverse the arginine deficiency state in the tumor microenvironment.

### 3.4 Proline

Proline metabolism regulates the functional state and spatial distribution of immune cells in the tumor immune microenvironment (TME) through two main pathways: synthesis (mediated by PYCR1/2) and degradation (mediated by PRODH). At the level of circulating immune cells, the abnormal activation of proline metabolism in tumor cells constitutes a systemic immune disturbance through the secretion of metabolites and the release of inflammatory factors. In clear cell renal cell carcinoma (ccRCC), hypoxia-induced high expression of PYCR1 not only enhances the tumor’s own proline synthesis but also promotes the secretion of proline-rich collagen precursors into the blood, indirectly affecting the bone marrow output and phenotypic polarization of circulating monocytes and neutrophil precursors (82). More importantly, the overexpression of PRODH in lung cancer triggers an ROS burst, which leads to the nuclear translocation of IKKα and upregulates the transcription and release of inflammatory factors such as CXCL1, LCN2, and IL-17C - these factors not only recruit immature myeloid cells, but also directly act on peripheral T cells and NK cells: CXCL1, by binding to CXCR2, weakens the chemotactic response of effector T cells to CXCR3 ligands (such as CXCL9/10), and hinders their homing to the tumor (83). While the cyclic P5C (generated by PRODH and released in exosomes or free form) is efficiently taken up by peripheral MDSCs. By inhibiting mitochondrial complex IV and activating the NLRP3 inflammatory body, it significantly enhances the expression levels of ARG1, iNOS and PD-L1, and increases its immunosuppressive potential (84). Meanwhile, P5C exhibits highly selective toxicity towards circulating NK cells – it targets the mitochondrial complex III, triggering a mtROS storm and ATP depletion, resulting in severe impairment of NK cell degranulation ability and IFN-γ secretion, while the expression of surface activating receptors (NKG2D, NKp30) remains unaffected, presenting a typical “functional exhaustion rather than inactivation” state (85). It is worth noting that in the peripheral blood of advanced patients, the CD56bright NK cell subset is often accompanied by a compensatory upregulation of PYCR2, suggesting that this subset may utilize the proline synthesis pathway to regenerate NADPH to resist oxidative stress, thereby obtaining immunoregulatory and even inhibitory functions, constituting a new type of circulating immunosuppressive NK cells (86).

At the level of tumor-infiltrating immune cells, the disruption of proline metabolism further amplifies the immunosuppressive effect locally. The excessive collagen deposition driven by PYCR1 significantly increases the rigidity of the extracellular matrix (ECM), not only forming a physical barrier to hinder T cell infiltration, but also promoting the polarization of tumor-associated macrophages (TAM) towards the M2 type through the DDR2-YAP/TAZ-LOXL2 axis, and enhancing the adhesion, retention, and PD-L1 expression of MDSC around blood vessels and at the tumor margin (87). Meanwhile, tumor-derived P5C is abundantly enriched in the TME and becomes a core messenger molecule connecting the dysfunction of various immune cells: after being taken up by infiltrating MDSCs, it strengthens their ROS-NF-κB-IL-10 pathway, and collaborates with TGF-β to form a potent immunosuppressive loop (84). When taken up by Tregs, it activates mTORC1-SREBP1, stabilizes FOXP3 expression and enhances lipid synthesis, significantly increasing its inhibitory strength and TME retention ability (82). For infiltrating CD8^+^ T cells, P5C enters the cells through the SLC36A1 transporter. On one hand, it inhibits mitochondrial complex III, causing persistent energy depletion and an apoptotic tendency. On the other hand, it leads to a deficiency of proline, specifically blocking the translation synthesis of Granzyme B and Perforin, thereby retaining the TCR recognition ability while losing the cell-killing function (85). For the infiltrating NK cells, P5C also inhibits the accumulation of mtROS and the collapse of mitochondrial membrane potential through complex III, resulting in a significant decrease in the proportion of IFN-γ^+^/CD107a^+^ cells, and accelerating their apoptosis and clearance within the tumor (86). Single-cell multi-omics studies further confirmed that in the tumor microenvironment (TME) of renal and prostate cancer patients with high expression of PYCR1/PRODH, the mitochondrial respiratory chain genes (such as NDUFA4, UQCRB, COX7A2L) of CD8^+^ T cells and NK cells were generally downregulated, while the proline metabolism-related genes (PYCR1, ALDH18A1, PRODH) and antioxidant genes (SOD2, PRDX1) in MDSC and Treg were significantly upregulated, confirming the central role of this metabolic axis in shaping the heterogeneity of immune cell functions.

In summary, proline metabolism is not merely a passive indicator of tumor malignancy but is an active driver in constructing the dual barriers of “systemic immune insufficiency” and “local immune paralysis”. Its effects on circulating MDSC and NK cells determine the “starting threshold” of anti-tumor immune responses, while its targeted regulation of tumor-infiltrating MDSC, Treg, CD8^+^ T, and NK cells directly sets the “terminal upper limit” for the effectiveness of immunotherapy. Therefore, targeting proline metabolism - for example, by jointly inhibiting PYCR1 to reduce P5C supply, using P5C dehydrogenase (such as P5C dehydrogenase) to neutralize its toxicity, or developing SLC36A1 antagonists to block P5C endocytosis - is expected to simultaneously restore the functional integrity of T cells and NK cells from both systemic and local perspectives, providing an original metabolic immunotherapy intervention strategy to overcome ICI resistance.

### 3.5 Serine

Serine metabolism serves as a central metabolic hub integrating glycolysis, one-carbon (1C) unit metabolism, and redox homeostasis. In the tumor immune microenvironment (TIME), it exerts highly cell-type–specific and spatially constrained regulatory functions. Accumulating evidence indicates that serine is far more than a proteinogenic amino acid: its derivatives—including glycine, S-adenosylmethionine (SAM), and 1C units carried by tetrahydrofolate (THF)—are indispensable for immune cell proliferation, differentiation, epigenetic reprogramming, and maintenance of effector functions. In the peripheral circulation, naïve T cells, natural killer (NK) cells, and monocytes rely critically on exogenous serine to sustain basal mitochondrial respiration, glutathione (GSH) biosynthesis, and DNA replication. For instance, Kishton et al. demonstrated that serine deprivation potently arrests naïve CD8^+^ T cells at the G_1_-to-S transition—a defect refractory to rescue by pyruvate or α-ketoglutarate—underscoring serine’s non-redundant role as a nucleotide precursor (88). Similarly, Zhang et al. showed that serine uptake via the transporter SLC1A4 (ASCT1) is essential for NK cell function: genetic ablation of SLC1A4 reduced IFNγ production by >50%, concomitant with diminished mTORC1 signaling and loss of mitochondrial membrane potential (89).

In stark contrast, this metabolic support system is fundamentally subverted within the tumor parenchyma. PHGDH-overexpressing cancer cells—including those in breast cancer, melanoma, and non-small cell lung cancer—hyperactivate the serine synthesis pathway (SSP), diverting substantial amounts of the glycolytic intermediate 3-phosphoglycerate (3-PG) to *de novo* serine production. This results in profound local depletion of extracellular serine—concentrations plummeting to 20–40 μM, well below physiological plasma levels (120–150 μM)—thereby imposing selective metabolic stress on tumor-infiltrating lymphocytes (TILs) (90, 91).

Under these conditions, tumor-infiltrating CD8^+^ T cells exhibit impaired serine utilization due to SLC1A4 internalization/degradation and downregulation of mitochondrial serine hydroxymethyltransferase 2 (SHMT2). This triggers a cascade of functional impairments: SAM depletion leads to global reductions in histone H3K4me3 and H3K27ac marks, specifically compromising chromatin accessibility at promoters of key effector genes (IFN-γ, GZMB, TNF); concurrently, insufficient GSH synthesis exacerbates intracellular ROS accumulation, causing oxidative inactivation of proximal TCR signaling molecules (e.g., LCK, ZAP70) and activating the ATF4–CHOP integrated stress response, ultimately promoting apoptosis (92, 93).

Critically, immunosuppressive cell populations display heightened metabolic plasticity under serine restriction. Regulatory T cells (Tregs) upregulate the glycine cleavage system (GLDC) to salvage 1C units from glycine and sustain FOXP3 expression via fatty acid oxidation. Meanwhile, M2-polarized tumor-associated macrophages (TAMs) synergize with HIF-1α stabilization under low-serine conditions to enhance expression of immunosuppressive mediators—including ARG1 and VEGF—thereby reinforcing an immune-tolerant niche (94, 95).

Clinical correlative data further validate the translational relevance of this axis: In melanoma patients, high intratumoral PHGDH expression strongly correlates with reduced CD8^+^ T cell infiltration (r = −0.62, p < 0.001) and significantly shorter progression-free survival following anti–PD-1 therapy (96).

Collectively, serine metabolism is not a passive nutrient supply route but rather a bona fide “metabolic checkpoint” governing immune cell fitness and functional polarization within the TIME. Therapeutic strategies targeting tumor-intrinsic SSP—such as PHGDH inhibitors (e.g., NCT-503)—or selectively augmenting serine utilization in TILs—via nanocarrier-mediated delivery of SHMT2 mRNA—represent promising avenues to overcome immune checkpoint blockade resistance (97) (**Table 2**).

**Table 2:** The effects of five amino acids on the functions of immune cells, as well as the related inhibitors at the experimental or clinical stage.

Amino acid	The impact on the function of immune cells	Related experimental or clinical-stage inhibitors
Glutamine	Tumors competitively deplete the microenvironmental glutamine, resulting in limited energy and biosynthesis for T cells, inhibiting their activation, proliferation, and Th1/Th17 differentiation, and promoting Treg differentiation; M2-type macrophages rely on high glutamine breakdown to maintain ARG1 activity and immunosuppressive function; GLS2 selective inhibition can promote the transformation of macrophages to the M1-like phenotype	Elaglenastat (CB-839, GLS1 inhibitor) has entered the II/III phase clinical trials for combination with PD-1 antibodies for NSCLC, renal cancer, etc. (such as NCT03207145); LAT1 inhibitor JPH203 is in the experimental stage; LAT2 genetic or pharmacological inhibition can restore macrophage phagocytic function and enhance anti-tumor immunity
Serine	Supporting the proliferation of T/NK cells, the expression of effector molecules (IFNγ, GzmB), epigenetic activation (H3K4me3/H3K27ac), and redox homeostasis (GSH synthesis); The tumor-depleted microenvironment depletes serine, specifically damaging the function of CD8^+^ T cells, but sparing/enabling Tregs and M2-TAMs, thereby exacerbating immunosuppression	PHGDH inhibitors (such as NCT-503 and CBR-5884) are in the preclinical research stage
Proline	When tumor-derived P5C is taken up by MDSC, Treg, CD8^+^ T cells, and NK cells: MDSC: ↑ ARG1/iNOS/PD-L1, enhancing immunosuppression; Treg: ↑ mTORC1-SREBP1, stabilizing FOXP3, enhancing inhibitory function; CD8^+^ T: ↓ Granzyme B/Perforin, losing killing ability but retaining recognition ability; NK: mtROS storm → ATP depletion → impaired degranulation and IFN-γ secretion	PYCR1 inhibitors (such as compound 6, derivatives of CB-5083) are in the preclinical stage; P5C dehydrogenase activators or SLC36A1 antagonists represent new strategies
Arginine	Arginine depletion leads to: T cells: impaired TCR signaling, NO-mediated apoptosis, cell cycle arrest; NOS uncoupling produces RNS that damages function; Macrophages: promote M2 polarization; MDSC: enhance inhibitory function	CB-1158 (pan-arginase inhibitor): Phase I clinical trial results showed that it could restore the activity of CD8^+^ T cells, and objective remission was observed
Tryptophan	Lack of tryptophan → Activation of the GCN2-eIF2α-ATF4 pathway → T cells: G1 phase arrest, impaired Th17 differentiation, enhanced Treg differentiation; AhR activation → Broad immunosuppression	Epacadostat (IDO1 inhibitor): Failed in Phase III ECHO-301 trial; Currently shifting to dual-target (IDO1/TDO2) inhibitors or AhR antagonists (IK-175)

## 4 Discussion

The dual role of amino acid metabolism—as both a driver of tumor-intrinsic anabolic fitness and a master regulator of immune cell function—has emerged as a central axis in cancer immunobiology. Tumor cells rewire their metabolic networks to hyper consume key amino acids (e.g., glutamine, serine, arginine, tryptophan, proline) not only to fuel biosynthesis and redox homeostasis but also to actively sculpt an immunosuppressive microenvironment. This is achieved through coordinated upregulation of nutrient transporters (e.g., ASCT2/SLC1A5, LAT1/SLC7A5), induction of catabolic enzymes (e.g., IDO1, ARG1, GLS), and secretion of immunomodulatory metabolites (e.g., kynurenine, ornithine, reactive nitrogen species). Crucially, these same amino acids are indispensable for effector T cell activation, clonal expansion, and cytotoxic function—creating a fierce metabolic competition within the tumor microenvironment (TME). When tumor cells gain dominance in this competition—via tryptophan depletion–induced GCN2-mediated T cell cycle arrest, or arginine scarcity–driven impairment of TCR signaling and nitric oxide synthesis—they effectively suppress anti-tumor immunity while promoting the expansion and function of regulatory T cells (Tregs) and myeloid-derived suppressor cells (MDSCs). Thus, amino acid metabolism serves not merely as a housekeeping process but as a functional interface linking oncogenic reprogramming to immune evasion.

This mechanistic understanding has directly inspired a new class of immunometabolic therapeutics. Preclinical models robustly demonstrate that pharmacologic inhibition of glutaminase (e.g., telaglenastat/CB-839), arginase (e.g., CB-1158), or amino acid transporters (e.g., ASCT2, LAT1) can reverse T cell dysfunction, deplete immunosuppressive populations, and—most notably—synergize potently with immune checkpoint inhibitors (ICIs), particularly anti-PD-1/PD-L1 antibodies. Such combinations hold particular promise for overcoming primary resistance to ICIs, underscoring the therapeutic value of co-targeting metabolic and immune checkpoints.

Yet, the stark disconnect between compelling preclinical synergy and clinical efficacy was soberingly exemplified by the phase III ECHO-301 trial, in which the IDO1 inhibitor epacadostat failed to improve progression-free or overall survival when added to pembrolizumab in advanced melanoma (98). This pivotal failure exposed fundamental gaps in our translational framework—and highlighted four interrelated challenges that must be systematically addressed:

1. Intratumoral metabolic heterogeneity: Expression and activity of metabolic enzymes (e.g., IDO1, ARG1, GLS) vary markedly across tumor regions and between patients (99). Unselected patient populations—particularly those lacking functional target engagement (e.g., low intratumoral kynurenine/tryptophan ratio or negligible enzyme expression) (100)—dilute treatment effect, emphasizing the urgent need for functional biomarker–guided stratification, rather than mere genomic or IHC-based selection (101).
2. Pathway redundancy and compensatory adaptation: Biological systems rarely rely on single nodes. Inhibition of one pathway frequently triggers rapid upregulation of alternatives—for instance, suppression of ASCT2-mediated glutamine uptake may be bypassed by increased expression of sodium-coupled neutral amino acid transporters (SNATs) or enhanced *de novo* glutamine synthesis via glutamine synthetase; similarly, IDO1 blockade can induce compensatory expression of IDO2 or hepatic/brain-localized tryptophan 2,3-dioxygenase (TDO2), sustaining systemic kynurenine production and immunosuppression (102).
3. Adaptive metabolic rewiring: Tumor cells exhibit profound metabolic plasticity under selective pressure. Glutamine-deprived cells may shift toward macropinocytic protein scavenging or increase reliance on alternative fuels—including lactate, fatty acids, or even extracellular vesicles—thereby evading monotherapeutic metabolic blockade (103).
4. A narrow therapeutic window: Many amino acid metabolic pathways are nonredundant in healthy physiology: arginine is essential for hepatic ammonia detoxification (urea cycle), tryptophan for serotonin and NAD^+^ biosynthesis, and methionine/folate for one-carbon metabolism and epigenetic regulation (104). Systemic inhibition thus carries substantial on-target toxicity risks—including neurotoxicity, gastrointestinal barrier disruption, or immune dysregulation—limiting dose intensity, duration, and tolerability (105).

The epacadostat experience therefore serves not as a refutation of the immunometabolic hypothesis, but as a critical calibration point: it underscores that target validation, even at the molecular and preclinical level, is insufficient without contextual understanding of dynamic feedback, tissue-specific biology, and functional pathway engagement in patients (106).

To translate immunometabolic targeting into durable clinical benefit, future strategies must pivot from broad-spectrum, single-node inhibition toward precision metabolic medicine. This entails three complementary pillars:

First, biomarker-driven patient selection, leveraging functional readouts such as plasma kynurenine/tryptophan ratio (107), tumor GLS or SLC7A5 expression (by spatially resolved proteomics (108) or PET-based glutamine analog tracers) (109), or integrated metabolic gene signatures;

Second, rational polypharmacology, including simultaneous inhibition of nonredundant nodes (e.g., dual IDO1/TDO2 blockade; co-targeting glutamine and serine metabolism via PHGDH inhibition) (110);

Third, context-aware modulation, exploiting differential metabolic dependencies—such as dietary methionine restriction or PYCR1 inhibition in proline metabolism—to selectively impair tumor proliferation while preserving T cell fitness. Emerging approaches—including localized delivery (e.g., nanoparticle-encapsulated inhibitors), intermittent dosing schedules, or microbiome-mediated modulation of tryptophan catabolism—may further widen the therapeutic index by decoupling systemic toxicity from local immunorestoration (111).

In conclusion, amino acid metabolism represents a linchpin connecting tumor cell autonomy and immune surveillance failure. Its therapeutic targeting demands a paradigm shift: away from static, monolithic inhibition toward a dynamic, ecosystem-level understanding of the TME—integrated across genomics, single-cell immunoprofiling, spatial metabolomics, and real-time *in vivo* metabolic tracing. Only through such multidimensional interrogation can we design smarter, safer, and truly personalized “metabolism–immune” combination regimens—ushering in the next generation of precision immuno-oncology.
