# Correction: The role of abnormal amino acid metabolism in the occurrence and development of tumors

**DOI:** 10.3389/fonc.2026.1824927

**Published:** 2026-04-07

**Authors:** Yuting Zhang, Honglin Chen

**Affiliations:** 1 Department of Pharmacy, No. 87 Zhongda Hospital Eastsouth University, Nanjing, Jiangsu, China; 2 Department of Pharmacy, Jingjiang Traditional Chinese Medicine Hospital, Jingjiang, Jiangsu, China

**Keywords:** amino acid, clinic, immunity, metabolism, tumor

Author “Honglin Chen” was erroneously omitted as equal contributing author. The following statement was erroneously omitted for the authors: “These authors have contributed equally to this work and share first authorship.”

The original version of this article has been updated.

